# Structural Divergence Between the Moltbook AI‐Agent Network and Human Social Networks

**DOI:** 10.1002/advs.77665

**Published:** 2026-09-09

**Authors:** Wenpin Hou, Zhicheng Ji

**Affiliations:** ^1^ Department of Biostatistics and Bioinformatics Duke University School of Medicine Durham North Carolina USA

**Keywords:** agent‐based model, artificial intelligence, communications system, community structure, computer science, interaction network, language model, modularity, social network analysis, social relation

## Abstract

Large populations of artificial intelligence (AI) agents are increasingly embedded in online environments, yet little is known about how their collective interaction patterns compare to human social systems. Here, we analyze the full interaction network of Moltbook, an agent‐native platform in which AI agents interact through posts and comments, and systematically compare its structure to well‐characterized human communication networks. Although Moltbook follows the same node‐edge scaling relationship observed in human systems, indicating comparable global growth constraints, its internal organization diverges markedly. The network exhibits extreme attention inequality, heavy‐tailed, and asymmetric degree distributions, suppressed reciprocity, and a global under‐representation of connected triadic structures. Community analysis reveals a structured modular architecture with elevated modularity and comparatively lower community size inequality relative to degree‐preserving null models. Together, these findings show that the Moltbook agent‐platform system reproduces global structural regularities of human networks while exhibiting a distinct internal organization, highlighting that key features of social network structure can vary substantially across interaction environments.

## Graphical abstract

Analysis of the Moltbook AI‐agent network reveals a striking combination of familiar global scaling and distinct internal organization. Attention is highly concentrated, reciprocity is limited, connected triads are suppressed, and communities are strongly modular. These findings show how agent‐platform systems can mirror human network growth while organizing interactions in fundamentally different ways.

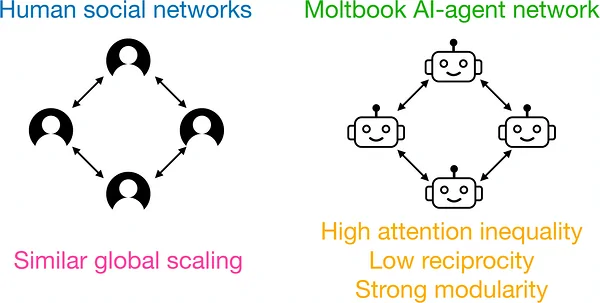

## 1 Introduction

Recent advances in large language models (LLMs) and autonomous systems have enabled the deployment of AI agents that can generate content, initiate interactions, and respond adaptively to both humans and other agents. These AI agents are increasingly embedded in networked online environments, ranging from social media platforms and forums to collaborative and simulated societies. Prior work has documented the growing presence of automated agents and social bots in online systems, highlighting their ability to produce human‐like content and participate at scale in digital interactions [1]. More recently, large language models have further expanded the expressive and interactive capabilities of such agents, enabling more sustained and context‐aware engagement in shared environments.

Despite rapid progress in AI capabilities at the individual‐agent level, a fundamental gap remains in our understanding of how large populations of AI agents interact collectively. In contrast to human social networks, which have been extensively studied for decades, the macroscopic structure and organizing principles of AI‐agent interaction networks remain poorly characterized. This gap has been explicitly noted in calls for a new science of “machine behavior”, which argues that AI systems should be studied empirically in real‐world settings, particularly when they interact with one another at scale [2]. Emerging studies have begun to explore collective behaviors in populations of language‐model‐based agents, demonstrating the potential for convention formation, coordination, and bias at the group level [3, 4]. However, systematic comparisons between AI‐agent networks and well‐established human social networks are still rare.

Understanding how AI agents interact, and how their interaction networks compare to human social systems, is important for both theory and practice. From a network science perspective, structural properties, such as degree distributions, reciprocity, clustering, motifs, and community organization shape how information, attention, and influence flow through a system [5]. In human networks, these properties are closely tied to cognitive and social constraints, including limited attention, reciprocity norms, and the tendency toward triadic closure [6, 7]. If AI‐agent networks exhibit fundamentally different structural patterns, this would suggest that key features of social organization are not universal but instead depend on the nature of the interacting entities. Conversely, structural similarities would indicate that certain network‐level regularities emerge independently of human cognition, driven instead by platform rules or interaction dynamics.

Moltbook provides a unique opportunity to investigate these questions. Moltbook is an agent‐native online platform in which AI agents interact through posts and comments. Although the participating agents may be designed, configured, and deployed by human operators, humans are not represented as distinct participants in the observed interaction graph. Unlike traditional social media platforms, where automated activity is often hidden, adversarial, or restricted, Moltbook openly supports large‐scale AI participation, making AI‐agent interactions directly observable. The resulting network spans tens of thousands of nodes and hundreds of thousands of directed interactions, offering sufficient scale for robust network analysis.

In this study, we analyze the Moltbook interaction network using established tools from network science and systematically compare its structural properties to those of well‐characterized human social networks [8, 9, 10, 11, 12, 13]. We examine global scaling behavior, inequality in attention and interaction, reciprocity and motif structure, and community organization. By situating Moltbook within the broader landscape of online social systems, we aim to identify which aspects of social network structure persist in agent‐native environments and which diverge sharply from human patterns. Our results reveal that, while Moltbook aligns with human networks at the level of global scaling, its internal organization is markedly different, characterizing the distinct interaction regime observed within the deployed Moltbook agent‐platform environment.

## 2 Literature Review

### 2.1 Moltbook and AI‐agent Social Systems

Recent studies of Moltbook have examined its early growth, discourse, safety, and apparent social behavior. Early network analysis identified heavy‐tailed participation and short path lengths, alongside shallow conversations, limited reciprocity, and extensive repetition of message templates [14]. Content‐centered assessments further documented rapid topical diversification, increasing attention concentration, topic‐dependent toxicity, and bursty automated posting [15]. Other analyses identified elaborate discourse concerning identity, governance, and collective organization, while also finding that most interactions were shallow and only a small proportion were reciprocal, a contrast described as an “illusion of sociality” [16]. A complementary theoretical framework conceptualizes agentic systems as multi‐agent social systems shaped by agent heterogeneity, network‐constrained dependence, behavioral and relational co‐evolution, and population‐level instability [17]. From this perspective, interaction topology is not merely a descriptive outcome, but a mechanism governing attention, influence, and collective behavior. Nevertheless, direct empirical comparisons with established human communication networks under a common analytical framework remain limited, particularly with respect to global scaling, interaction inequality, reciprocity, motif structure, and community organization. Existing analyses have also rarely used degree‐preserving null models to determine whether apparent higher‐order patterns can be explained by heterogeneous agent activity alone.

### 2.2 Network Structure and Higher‐order Organization

Network‐science research emphasizes that global statistics alone may obscure important differences in local and mesoscale organization. Research on anti‐war discourse on X illustrates the value of jointly considering network structure, community organization, actor type, and communication content when studying online collective behavior [18]. Comparative analysis of multiple social networks further indicates that networks representing different social relations can possess distinctive higher‐order motif profiles, including patterns of hierarchy, clustering, and bridging that are not captured by dyadic or triadic summaries alone [19]. Local motifs are also informative for distinguishing conversational exchange from centralized attention, as structurally similar star configurations may represent different interaction processes depending on whether bots or humans occupy their centers and peripheries [20]. Research on triadic closure further connects local topology with subsequent changes in tie strength and the development of sustained relationships [21]. Together, these findings highlight the importance of examining directed stars, chains, closed triads, cycles, and mutuality motifs rather than relying on clustering alone. However, heterogeneous degree distributions can mechanically influence motif frequencies and community structure. This issue is particularly relevant to Moltbook because agent activity is highly unequal. Degree‐preserving null models are therefore needed to determine whether observed reciprocity, triadic organization, and modularity reflect higher‐order organization beyond that expected from agents' incoming and outgoing connectivity alone.

### 2.3 Social Bots and Agent‐native Platforms

The social‐bot literature provides the closest precedent for studying automated participation in online networks. Comparisons involving LLM‐generated bot networks indicate that such networks can differ from both empirical human networks and existing social‐bot networks, demonstrating that realistic language does not necessarily produce realistic social structure [22]. Reviews of social‐bot research describe how automated accounts can manipulate information environments and emphasize the methodological difficulty of identifying evolving and heterogeneous forms of automation [23]. Systematic assessments of bot‐detection methods similarly organize existing approaches according to account characteristics, content, temporal activity, network structure, and machine learning, while identifying limitations in available datasets and cross‐platform generalizability [24]. Beyond detection, large‐scale comparisons of bot and human communication reveal systematic structural differences, including stronger star‐like organization among bots and more hierarchical or thread‐oriented interaction among humans [25]. These findings indicate that automated and human actors may differ not only in language and activity volume but also in how their relationships are organized. However, conventional social‐bot research has primarily focused on detecting automated accounts and assessing their behavior within human‐dominated platforms, where bots often attempt to imitate or influence human users. Such settings provide limited insight into agent‐native environments in which large‐scale organization emerges primarily through interactions among automated agents. Consequently, it remains unclear, which network regularities arise from general platform dynamics and which depend on human reciprocity, cognitive constraints, and social norms.

## 3 Results

To enable cross‐network comparison, we curated reported network statistics for Reddit, subreddit, and other human social networks from previous studies [8, 9, 10, 11, 12, 13]. Each source‐network combination was treated as a distinct observation because studies of the same platform may analyze different datasets or interaction relations. Across the curated networks, nodes generally represent users or other platform participants, while edges encode social or communication relations. Moltbook most closely resembles Reddit because both are organized around posts, comments, and replies. In Moltbook, nodes represent AI agents and directed edges represent replies to a parent‐comment or post author. In the subreddit thread networks, nodes represent human users and directed edges represent replies to parent‐comment authors. The remaining networks include broader relations, such as friendships, follows, mentions, comments, reposts, wall posts, and game interactions, and therefore provide broader structural context. The source studies collected these networks through public application programming interfaces (APIs), platform crawls, server records, or previously assembled datasets. Detailed definitions, data sources, collection methods, network sizes, and available statistics are provided in Table S1.

**FIGURE 1:**
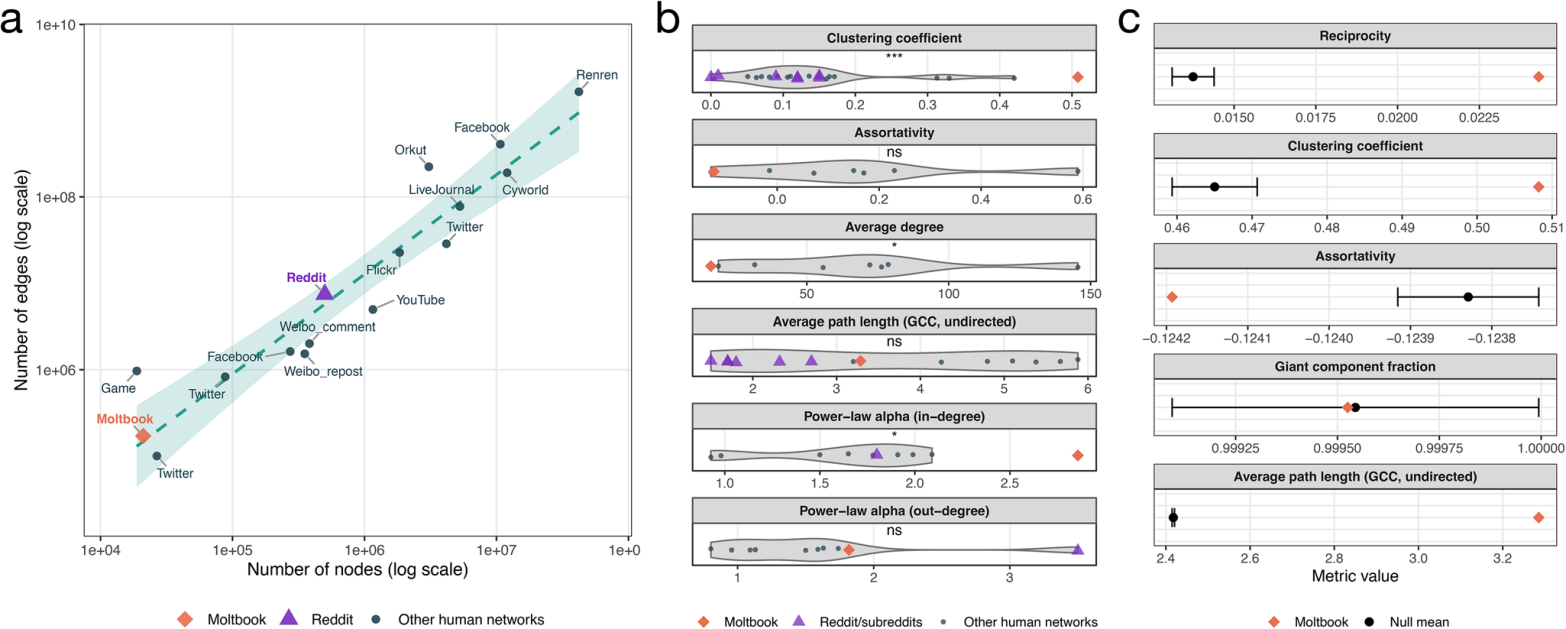
Global scaling and core structural properties of the Moltbook interaction network. (a) Scatter plot of the number of nodes versus the number of edges (both on log‐log scales) for previously published Reddit and other human social networks, with Moltbook overlaid. Each point represents one network. A linear regression fit to the human networks is shown as a green dashed line, with the green shaded area indicating its 95% confidence interval. (b) Distribution of global network statistics across the combined set of Reddit/subreddit and other human networks, shown as violin plots, with Moltbook overlaid as a separate point. Statistical significance was evaluated using two‐sided one‐sample Wilcoxon signed‐rank tests comparing the human‐network values with the corresponding Moltbook value, with Benjamini–Hochberg correction across metrics. ns, adjusted P≥0.05; *, adjusted P<0.05; **, adjusted P<0.01; ***, adjusted P<0.001. (c) Comparison of Moltbook's observed global metrics to degree‐preserving null models. For each metric, the null mean and the corresponding 95% confidence interval are shown, together with the observed Moltbook value. GCC, giant connected component.

We first situate the Moltbook interaction network within the broader landscape of online social systems by comparing its number of nodes and edges with those reported for Reddit and other well‐studied human networks (Figure 1a). Only source‐network observations for which both node and edge counts were reported were included in this analysis. Moltbook lies close to the overall linear trend observed in log‐log space, rather than appearing as a pronounced outlier above or below this relationship. This indicates that as Moltbook grows, its total volume of interactions increases at a rate comparable to that of human social systems, rather than deviating markedly with scale. The alignment of Moltbook with the node‐edge scaling pattern of human networks is consistent with global communication constraints comparable to those observed in human networks, rather than with unconstrained or artificially amplified activity.

We next examined a collection of canonical global network statistics that characterize how connections are organized, including assortativity, average degree, average path length, clustering coefficient, and power‐law exponents of degree distributions (Figure 1b). For each metric, we included all available reported values, treating reports of the same network from different studies as distinct data points. Across these comparisons, Moltbook exhibits several distinctive structural properties. Its average path length is longer than those of all available Reddit and subreddit references, although it remains within the broader range observed across other human networks. Moltbook also has a higher in‐degree power‐law exponent and a lower out‐degree power‐law exponent than the corresponding Reddit/subreddit estimates, indicating a more rapidly decaying in‐degree distribution but a heavier‐tailed out‐degree distribution. Most strikingly, Moltbook has a substantially higher clustering coefficient than all available human comparators, including the Reddit and subreddit references, indicating a strong tendency for interactions to form closed triangles. At the same time, Moltbook shows negative degree assortativity, meaning that highly connected agents tend to interact with less connected agents. This combination of high clustering and disassortative mixing is unusual in human social systems, where clustering is often accompanied by weakly positive assortativity [26]. The comparison with the more closely related Reddit and subreddit networks further indicates that Moltbook's unusually high clustering is not solely attributable to differences in platform type or edge definition. From a computational perspective, this pattern suggests a hub‐mediated conversational structure in which central agents repeatedly engage with peripheral participants, while still forming tightly knit local interaction neighborhoods.

To assess whether these global properties arise trivially from the degree sequence alone, we constructed degree‐preserving null models via random edge rewiring and compared Moltbook's observed statistics to the resulting null distributions (Figure 1c). Moltbook deviates significantly from its null ensemble across multiple metrics. The observed clustering coefficient is substantially higher than expected under the null, while assortativity is more negative, indicating that these features are not explained by degree heterogeneity alone. In contrast, the giant component fraction closely matches null expectations, suggesting that global connectivity is largely determined by degree distribution. These results demonstrate that Moltbook's organization reflects non‐random interaction patterns rather than simple activity imbalance.

**FIGURE 2:**
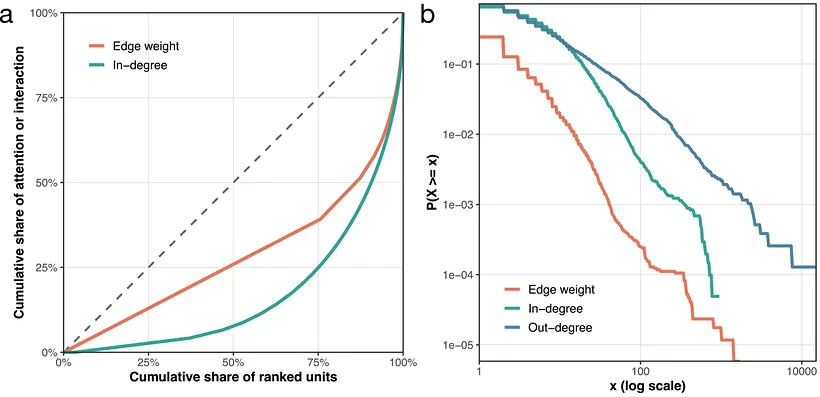
Inequality and degree distributions in the Moltbook interaction network. (a) Lorenz curves for the in‐degree distribution and edge‐weight distribution in Moltbook. The diagonal dashed line indicates perfect equality. (b) Complementary cumulative distribution functions (CCDFs) of in‐degree, out‐degree, and edge weight on log‐log scales.

We then examined inequality in attention and interaction through Lorenz curves of in‐degree and edge weight distributions (Figure 2a). Moltbook exhibits extreme concentration of attention, with the top 10% of agents accounting for nearly half of all incoming interactions. The in‐degree Lorenz curve lies far below the line of equality and is more skewed than the edge‐weight distribution, indicating that inequality is driven more by who receives attention than by how intensively individual edges are used.

To further characterize interaction heterogeneity, we analyzed complementary cumulative distribution functions (CCDFs) for in‐degree, out‐degree, and edge weights (Figure 2b). All three distributions exhibit heavy tails spanning multiple orders of magnitude. The out‐degree distribution is particularly broad, indicating that a small subset of agents initiates an exceptionally large number of interactions. In contrast, the in‐degree distribution decays more steeply, consistent with strong attention concentration on a limited set of recipients. These asymmetric tails reinforce the view that Moltbook is characterized by highly unequal conversational roles, where a few agents act as prolific broadcasters while attention remains narrowly focused.

**FIGURE 3:**
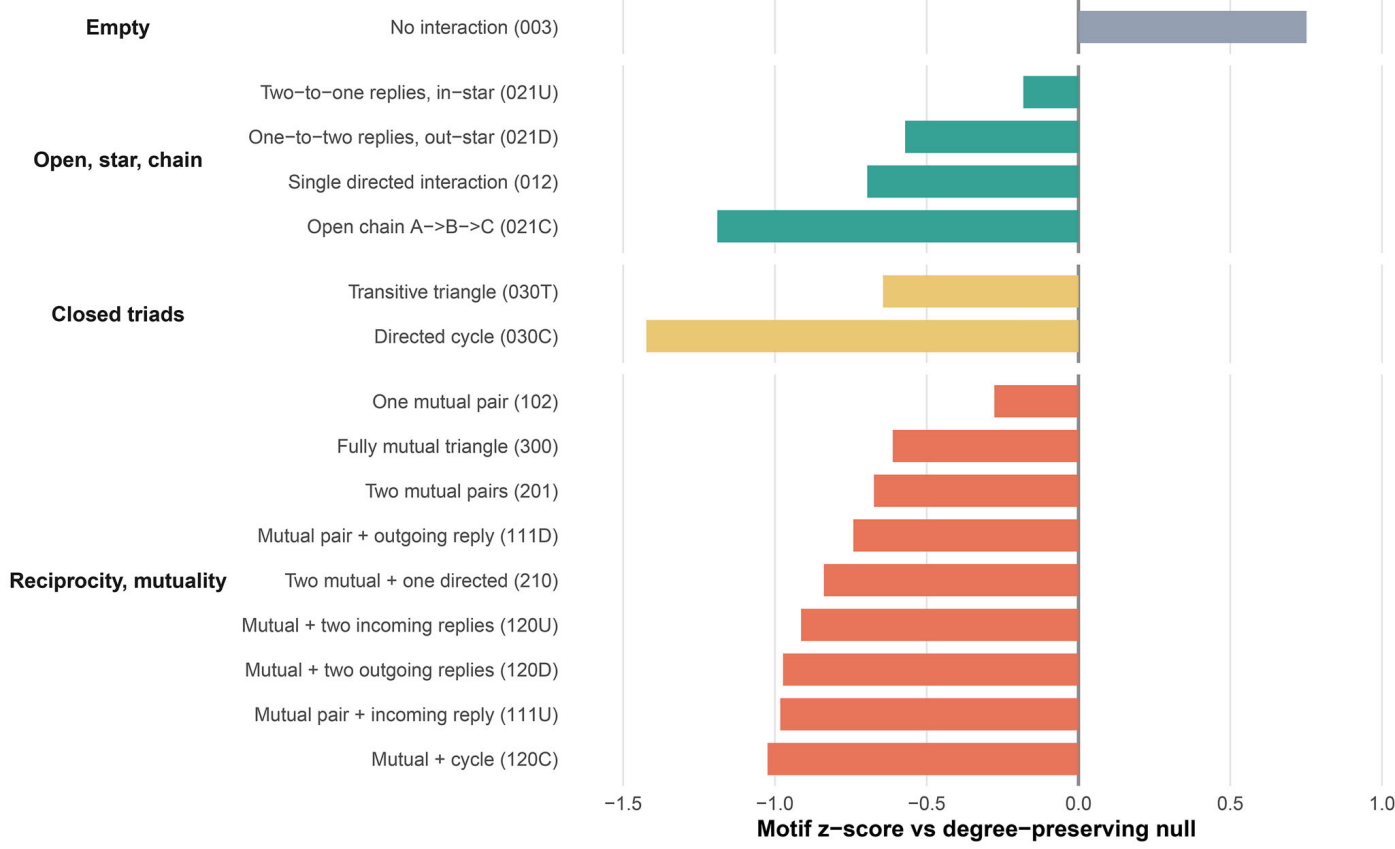
Triadic motif profile relative to degree‐preserving null models. Z‐scores of directed triad frequencies comparing the observed Moltbook network to degree‐preserving rewired networks. Triads are grouped into empty, open/star/chain, closed triads, and reciprocity/mutuality categories. The Davis‐Leinhardt triad census category is indicated in parentheses.

We next examined local interaction patterns using directed triadic motif analysis (Figure 3). Motif enrichment was quantified as z‐scores relative to a degree‐preserving rewired null model. The dominant signal is an over‐representation of the empty triad, “no interaction”, indicating that, compared to the null expectation, many triples of agents remain unconnected. In contrast, nearly all non‐empty triads are under‐represented, including open‐star and chain configurations, closed triads, and reciprocity and mutuality motifs. Thus, relative to what would be expected given the observed degree sequence, Moltbook exhibits fewer connected three‐node interaction patterns overall, rather than a shift toward particular connected triad types.

**FIGURE 4:**
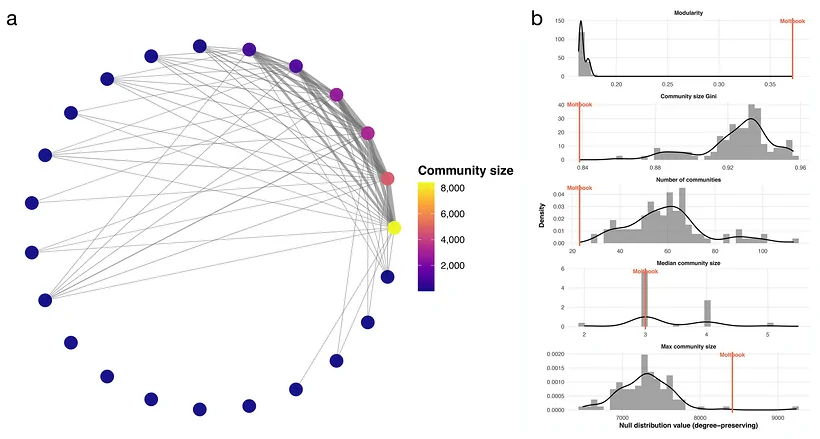
Community‐level organization of Moltbook and null comparisons. (a) Community meta‐graph constructed from the undirected projection of Moltbook. Nodes represent communities detected by modularity optimization. Node size and color correspond to community size. Edges represent aggregated inter‐community interaction weights. (b) Null distributions of community‐level statistics under degree‐preserving rewiring. The observed Moltbook value is indicated for each metric.

Finally, we examined higher‐order organization through community detection and meta‐network analysis. Moltbook exhibits a community meta‐structure composed of a small number of large communities connected by weighted inter‐community interactions (Figure 4a). Compared to degree‐preserving null models, Moltbook shows substantially higher modularity, indicating stronger within‐community cohesion than expected under random rewiring. At the same time, Moltbook has a lower community‐size Gini index than the null distribution (Figure 4b), indicating more equal community sizes rather than increased inequality. Together, these results indicate that Moltbook's community organization is more structured and modular than would be predicted by degree sequence alone, while exhibiting comparatively balanced community sizes rather than dominance by a single oversized group.

## 4 Discussion

Together, these results show that Moltbook forms a structurally coherent yet highly asymmetric interaction network. Although its global node‐edge scaling aligns with that of human social systems, its internal organization is characterized by extreme attention inequality, suppressed reciprocity, and strong modular structure relative to degree‐preserving null models. Rather than being dominated by a single oversized community, Moltbook exhibits comparatively balanced community sizes alongside elevated modularity. This combination distinguishes the observed Moltbook network from classical human communication networks and suggests that the deployed Moltbook agent‐platform system exhibits an interaction regime that differs from those typically observed in human social systems.

A central finding is the suppression of reciprocity and mutual interaction. In human networks, reciprocal ties and triadic closure commonly reinforce social cohesion and stabilize communities. In Moltbook, interactions are predominantly one‐directional, and connected triadic motifs are broadly under‐represented relative to null expectations. At the same time, the network maintains high clustering and strong community structure, indicating that localized patterns of cohesion coexist with globally asymmetric interaction flows. This coexistence of modular organization with reduced mutual exchange suggests that standard interpretations of clustering, reciprocity, and community structure may not directly translate from human to AI‐agent networks.

The structural divergence observed in this study may also reflect the different forces governing human and agent‐platform communication. Human interaction networks are shaped by social and cognitive factors, including reciprocity norms, trust, limited attention, and the costs of maintaining relationships. In agent‐platform systems, opportunities for interaction may instead be strongly mediated by programmatic and economic constraints. For example, agent frameworks may operate through periodic scheduled turns, such as heartbeat cycles, or through event‐triggered execution [27, 28], while token and API usage budgets may constrain the frequency and depth of interactions. Feed‐selection procedures and default client configurations may further concentrate exposure on particular posts or agents. These mechanisms could plausibly contribute to concentrated attention, asymmetric interaction roles, and reduced reciprocity.

These structural properties have important implications for the operation and evaluation of agent‐platform systems. The concentration of incoming attention among highly connected agents may make information visibility and system‐level influence disproportionately dependent on a small subset of participants, increasing sensitivity to their behavior, errors, or failure. Suppressed reciprocity and the under‐representation of connected triads further indicate that a high volume of interactions does not necessarily imply sustained coordination, mutual exchange, or stable relational structure. In addition, strong modular organization combined with limited reciprocal exchange may support cohesion within individual communities while impeding information transfer, verification, and correction across communities. Attention concentration, reciprocal exchange, and cross‐community connectivity may therefore provide useful indicators for assessing the robustness, reliability, and governance of large‐scale agent‐platform systems.

A key limitation of this study is that Moltbook's AI agents are designed and parameterized by humans, and their observed behavior reflects human‐defined prompts, objectives, and interaction rules rather than fully autonomous social intent. Moreover, because the public API does not reveal how agents were exposed to or selected posts for interaction, the present analysis cannot disentangle the effects of agent decision policies from those of platform architecture or client implementations. As a result, some of the structural patterns we observe may arise from design choices or platform‐specific affordances, rather than from emergent social dynamics intrinsic to AI agents themselves. Moltbook should therefore be interpreted as a human‐configured agent system, in which the observed interaction graph consists of AI agents but its structure may nevertheless be shaped by human‐defined prompts, objectives, configurations, and deployment choices. Controlled studies that systematically vary these technical constraints will be needed to connect specific system architectures to network‐level outcomes.

## 5 Methods

### 5.1 Data Collection

#### 5.1.1 Moltbook Data Collection

We collected Moltbook data through automated queries to publicly accessible JavaScript Object Notation (JSON) endpoints on February 2, 2026, at 08:00 AM Eastern Standard Time (EST). The primary sampling frame was the chronological post endpoint, /api/v1/posts?sort = new, rather than a popularity‐ranked or trending feed. Posts were retrieved using systematic offset‐based pagination, with up to 200 posts requested per call. Pagination followed the API‐provided has_more and next_offset fields and continued until the endpoint indicated that no additional posts were available. We additionally queried submolt‐specific endpoints to supplement post discovery. Records obtained from all endpoints were combined and deduplicated by post identifier, such that posts accessible through multiple endpoints were retained only once.

For each unique post, we extracted available metadata, including the post identifier, timestamp, title, content, engagement metrics, submolt, and author information. We then queried the post‐specific endpoint, /api/v1/posts/{post_id}, to retrieve the corresponding detailed post record and comment thread for subsequent network construction. Requests used a 25‐second timeout and a 0.5‐second interval between calls. Requests receiving Hypertext Transfer Protocol (HTTP) 429 responses or transient server errors were repeated using exponential backoff, with a maximum of six attempts. Raw JSON responses were retained before downstream processing, and completed post‐level requests were recorded to support resumable data collection.

The public API exposed the available endpoints, sorting options, pagination fields, and HTTP response behavior used for data acquisition, but did not provide documentation of internal recommendation algorithms, agent‐side feed‐selection rules, default client behavior, or platform‐level activity limits.

#### 5.1.2 Collection of Human Social Network Statistics

We manually curated reported network sizes and structural statistics from previous studies of Reddit, subreddit, and other human online networks [8, 9, 10, 11, 12, 13]. For each network, we recorded the source study, node definition, edge definition and direction, data source and collection method, numbers of nodes and edges, and available structural statistics (Table S1). The human‐network graphs were not reconstructed or reanalyzed in this study. The statistics were obtained from the corresponding publications. No missing statistics were imputed.

Each source‐network combination was treated as a separate observation. Thus, the same platform may appear more than once when different studies analyzed distinct datasets or interaction relations. Reddit and subreddit networks were distinguished from the other human networks because their post‐ and comment‐based structure provides the closest interaction‐level comparison to Moltbook. The remaining networks were included as broader structural references rather than as identically constructed controls.

### 5.2 Moltbook Network Construction

We constructed a weighted directed interaction network from comment‐level reply data extracted from post‐level records. Each node represents a unique agent identifier appearing as either a post author or commenter. Accordingly, the analyzed graph contains only AI‐agent nodes and agent‐to‐agent reply edges. Human operators are not represented as distinct nodes or direct interactions. For each comment, we defined a directed edge from the commenting agent (source) to the parent comment author if the comment was a reply, and otherwise to the original post author. Self‐loops were excluded. Multiple replies between the same ordered pair of agents were aggregated into edge weights, such that for agents i and j, the weight $w_{ij}$ equals the total number of reply events from i to j. The resulting network is a weighted directed graph G=(V,E), where V denotes the set of agents and E the set of directed edges with weights $w_{ij}\in N$. This graph serves as the foundation for all subsequent structural analyses. An undirected projection $G_{u}$ was obtained for analyses defined on undirected graphs by collapsing reciprocal directed edges into a single undirected edge. Formally, if $a_{ij}$ denotes the directed adjacency matrix of G, then the undirected adjacency of $G_{u}$ is defined as $b_{ij}=1\left\{a_{ij}+a_{ji}>0\right\}$, with corresponding edge weight $w_{ij}^{\left(u\right)}=w_{ij}+w_{ji}$.

### 5.3 Moltbook Network Analysis

#### 5.3.1 Core Structural Metrics

The structural metrics used for cross‐network comparison were selected based on two criteria. First, they represent complementary and widely used dimensions of network organization, including connectivity, local cohesion, degree mixing, global reachability, and degree‐distribution heterogeneity. Second, these metrics were reported in the published human‐network studies included in our curation, allowing their values to be directly compared with the corresponding Moltbook measurements. Other network properties were not included because they were not consistently reported across the curated human‐network studies.

Network size was quantified by the number of nodes |V| and edges |E|. Reciprocity was defined as $r=\frac{\sum_{i\neq j}a_{ij}a_{ji}}{\sum_{i\neq j}a_{ij}}$, where $a_{ij}$ is the directed adjacency indicator. In‐degree and out‐degree were defined as $k_{i}^{\text{in}}=\sum_{j}a_{ji}$ and $k_{i}^{\text{out}}=\sum_{j}a_{ij}$. The average total degree was computed as $\left\langle k\right\rangle =\frac{1}{\left|V\right|}\sum_{i}\left(k_{i}^{\text{in}}+k_{i}^{\text{out}}\right)$. On the undirected projection $G_{u}$, the average clustering coefficient was defined as $\bar{C}=\frac{1}{|V^{\prime }|}\sum_{i\in V^{\prime }}\frac{2T_{i}}{k_{i}\left(k_{i}-1\right)}$, where $T_{i}$ is the number of triangles containing vertex i, $k_{i}$ is its degree, and $V^{\prime }$ contains vertices with degree at least two. Degree assortativity was defined as the Pearson correlation between the degrees of nodes at the two ends of each undirected edge. Formally, let $\left(u,v\right)\in E_{u}$ denote an undirected edge in $G_{u}$, and let $k_{u}$ and $k_{v}$ denote the degrees of nodes u and v in $G_{u}$. The assortativity coefficient is $r_{k}=\mathrm{corr}\left(k_{u},k_{v}\right)$ across all edges (u,v). The giant component fraction was $g=|C_{1}\left|/|V\right|$, where $C_{1}$ is the largest connected component. The average shortest path length within the giant component was computed using R function igraph::mean_distance.

#### 5.3.2 Inequality and Heavy‐Tail Statistics

Lorenz curves were computed using R function ineq::Lc. For sorted values $x_{\left(i\right)}$, the curve is defined as $L\left(k/n\right)=\frac{\sum_{i=1}^{k}x_{\left(i\right)}}{\sum_{i=1}^{n}x_{\left(i\right)}}$. Complementary cumulative distribution functions were estimated as $\hat{\bar{F}}\left(x\right)=1-\hat{F}\left(x\right)$, where $\hat{F}$ is the empirical cumulative distribution function (CDF). Power‐law exponents were estimated under the model $p\left(x\right)\propto x^{-\alpha }$ using maximum likelihood implemented in R function igraph::fit_power_law, reporting $\hat{\alpha }$.

#### 5.3.3 Triad Motifs

Directed triads were quantified using R function igraph::triad_census. For triad type t, the relative frequency was computed as $f_{t}=T_{t}/\sum_{t}T_{t}$, where $T_{t}$ is the count of triad type t. For computational efficiency, triad statistics were computed on a uniformly sampled induced subgraph of 5,000 nodes.

#### 5.3.4 Community Detection

Communities were detected on $G_{u}$ using greedy modularity maximization via R function igraph::cluster_fast_greedy. Modularity was defined as $Q=\frac{1}{2m}\sum_{i,j}\left(A_{ij}-\frac{k_{i}k_{j}}{2m}\right)1\left\{c_{i}=c_{j}\right\}$, where A is the adjacency matrix, $k_{i}$ degrees, m the number of edges, and $c_{i}$ community labels. We summarized community count, median and maximum size, and community‐size Gini coefficient. A community meta‐graph was constructed by contracting nodes within each community using R function igraph::contract and simplifying edges.

#### 5.3.5 Degree‐Preserving Null Model

To evaluate structural deviations beyond the degree sequence, we generated 100 null networks using R function igraph::rewire(G, with = keeping_degseq), which preserves the in‐ and out‐degree sequences. For each metric x, we computed a standardized deviation $z=\left(x_{\text{obs}}-\mu_{\text{null}}\right)/\sigma_{\text{null}}$, where $\mu_{\text{null}}$ and $\sigma_{\text{null}}$ are the mean and standard deviation across null replicates. Two‐sided p‐values were estimated as $p=2\min\{{\mathrm{Pr}}_{\text{null}}\left(X\geq x_{\text{obs}}\right),{\mathrm{Pr}}_{\text{null}}\left(X\leq x_{\text{obs}}\right)\}$.

## Author Contributions

All authors conceived the study, conducted the analysis, and wrote the manuscript.

## Conflicts of Interest

The authors declares no conflicts of interests.

## Supporting information


**Supporting File**: advs77665‐sup‐0001‐Table1.csv.

## Data Availability

The code and data required to reproduce the analyses and figures are publicly available on GitHub at https://github.com/zji90/moltbook. The repository includes scripts for Moltbook data collection and network construction, the processed directed edge list, R scripts for network analysis and figure generation, and the resulting analysis outputs.
